# Lactic acid enhances vaginal epithelial barrier integrity and ameliorates inflammatory effects of dysbiotic short chain fatty acids and HIV-1

**DOI:** 10.1038/s41598-023-47172-y

**Published:** 2023-11-16

**Authors:** Ingrid Schwecht, Aisha Nazli, Biban Gill, Charu Kaushic

**Affiliations:** 1 Department of Medicine, McMaster University, Hamilton, ON Canada; 2 McMaster Immunology Research Center, Michael G. DeGroote Center for Learning and Discovery, McMaster University, Hamilton, ON Canada

**Keywords:** Immunology, Microbiology

## Abstract

The vaginal microenvironment is key in mediating susceptibility to sexually transmitted infections. A polymicrobial environment with reduced *Lactobacilllus spp.* is characteristic of vaginal dysbiosis, associated with increased production of several short chain fatty acids (SCFAs), vaginal inflammation and an increased risk of HIV-1 acquisition. In contrast, a eubiotic vaginal microbiome (VMB), dominated by *Lactobacillus spp.* correlates with increased production of lactic acid (LA), an acidic milieu and protection against HIV-1. Vaginal metabolites, specifically LA and SCFAs including butyric, succinic and acetic acids are associated with modulation of HIV-1 risk. We assessed the impact of combined and individual SCFAs and LA on vaginal epithelial cells (VK2) grown in air–liquid interface cultures. Treatment of VK2 cells with eubiotic SCFA + LA mixture showed increased epithelial barrier integrity, reduced FITC dextran leakage and enhanced expression of cell–cell adhesion proteins. Treatment with dysbiotic SCFA + LA mixture diminished epithelial barrier integrity, increased NFκB activation and inflammatory mediators: TNF-α, IL-6, IL-8 and RANTES. LA was found to be the primary contributor of the beneficial effects. Eubiotic SCFA + LA mixture ameliorated HIV-1 mediated barrier disruption and HIV-1 leakage, whereas dysbiotic SCFA + LA treatment exacerbated HIV-1 effects. These findings indicate a key role for LA in future prophylactic strategies.

## Introduction

Sexually transmitted infections (STI’s) inflict a disproportionate burden on women of reproductive age, increasing the incidence of infertility, pelvic inflammatory disease, and increased HIV-1 susceptibility^1^. Most commonly, infections in women begin in the lower female genital tract (FGT) following heterosexual transmission^2^. Consequently, protection in the vaginal microenvironment, both physiological and immunological, is critical for maintaining good reproductive health^3–5^. However, the mechanisms contributing to STI, and more specifically HIV-1 susceptibility remain poorly understood. Considering women account for over 50% of all new HIV-1 infections^6^, elucidating factors that influence the vaginal milieu are critical for improving reproductive health outcomes worldwide.

The microbial species present in the vaginal microbiome (VMB), and their relative production of metabolic biproducts are fundamental in modulating immunological responses at the mucosal surface in the lower FGT^7^. In particular, a eubiotic VMB dominated by *Lactobacillus spp*., coupled with a low microbial diversity has been associated with improved genital tract barrier and a reduction in HIV-1 susceptibility^8, 9^. In contrast, a polymicrobial VMB and decreased abundance of *Lactobacillus spp.* is characteristic of vaginal dysbiosis, that decreases the host’s mucosal barrier, thereby increasing the risk of infection^7^. Vaginal microbial communities, through the production of microbicidal compounds or by production of different metabolites, influence the function of the host physical and immunological defense system^9^. Moreover, certain metabolites produced under dysbiosis can cause inflammation, decrease cell–cell adhesion and compromise epithelial barrier integrity thereby altering the vaginal metabolome and impacting host-microbial interactions^1, 10^.

Bacterial vaginosis (BV) is considered one of the most common clinical conditions indicating dysbiosis in the FGT, currently affecting up to 30% of women^11^. Recent evidence suggests a 2.5-fold increase in HIV-1 susceptibility among those clinically diagnosed with BV^12^. Some bacterial species such as *Gardnerella vaginalis*, identified in 95% of BV cases^13–16^ ^13–18^, has been shown to increase ectocervical permeability and elevate levels of soluble epithelial E-cadherin, barrier related proteins and metabolites^17^, whereas *Lactobacillus crispatus* is known to protect the barrier and induce phenyllactic acid, an anti-microbial compound^15, 17^. *L. crispatus* has also been shown to mitigate the proinflammatory response induced in vaginal epithelial cells by BV-associated bacteria^14, 15, 17^. Similar trends in both epithelial barrier integrity and inflammatory profiles were observed when only the bacteria-free supernatants of *G. vaginalis* and *L. crispatus* were used, suggesting a role for microbially derived metabolites^13, 17^ However, the mechanisms by which the microbially derived metabolites interact, or elicit immunomodulatory effects to impact vaginal epithelial barrier integrity remains understudied^9, 16^. Characterization of the vaginal metabolome has revealed metabolic signatures of BV^19–25^, including increased concentrations of several short chain fatty acids (SCFAs) together with a decrease in lactic acid (LA)^23, 24, 26^.

Herein we explore the mechanisms by which the composition of microbial metabolites mimicking eubiotic and dysbiotic conditions in the vaginal tract can influence the vaginal epithelial barrier through expression of cell structural adhesion proteins and inflammatory mediators. Physiologically relevant concentrations of lactic acid (LA) and three SCFAs relevant to clinical BV including acetic acid (AA), succinic acid (SA) and butyric acid (BA), were used to model eubiosis and dysbiosis. Our results demonstrate the anti-inflammatory role of LA both individually and in the presence of SCFAs. Furthermore, we also demonstrate the specific protective role of LA on barrier integrity during both eubiotic and dysbiotic conditions. We also demonstrate the role of SCFAs on modulating the detrimental effects of HIV-1 on epithelial barrier. Modulating the vaginal microbiota and associated metabolites will play a key role in devising interventions to improve vaginal health of women^16^.

## Results

### Eubiotic metabolite treatment of vaginal epithelial cells enhances barrier integrity while dysbiotic metabolite treatment increases epithelial permeability

Previous studies have examined the inflammatory effect of several SCFAs on cervicovaginal epithelial cells^23, 24, 27^. However, their mechanistic role in mediating epithelial barrier integrity in the lower FGT has yet to be characterized. As such we investigated the impact of treatment of VK2 cells with SCFA + LA combinations that mimicked eubiotic and dysbiotic VMB conditions (Table 1). Barrier function was evaluated by measuring both transepithelial resistance (TER) and FITC-dextran leakage, indicative of epithelial monolayer integrity and cellular permeability respectively^28^. Our lab has previously demonstrated the efficacy of using the Air–Liquid interface (ALI) model of VK2 cell growth to study barrier integrity, as these cells exhibit both multilayer and tight junction formation^29–31^.

**Table 1:** Metabolite concentrations selected to model eubiotic and dysbiotic conditions in the vaginal microenvironment.

Metabolite	Eubiotic conditions		Dysbiotic conditions	
	Literature (mM)	Treatment (mM)	Literature (mM)	Treatment (mM)
Lactic acid	89–133^32^	100	> 20^33^11.5 −13.50^24^	20
Acetic acid	1.68–8.4^34^	4	40^35^	40
Succinic acid	0.00–0.61^33^	0.1	0.02–21.9^33^	10
Butyric acid	0.045–0.228^24^	0.1	2–4^27, 35^	2

Literature: mM concentrations from published work [references]; Treatment: mM concentration used in this study.

**Figure 1:**
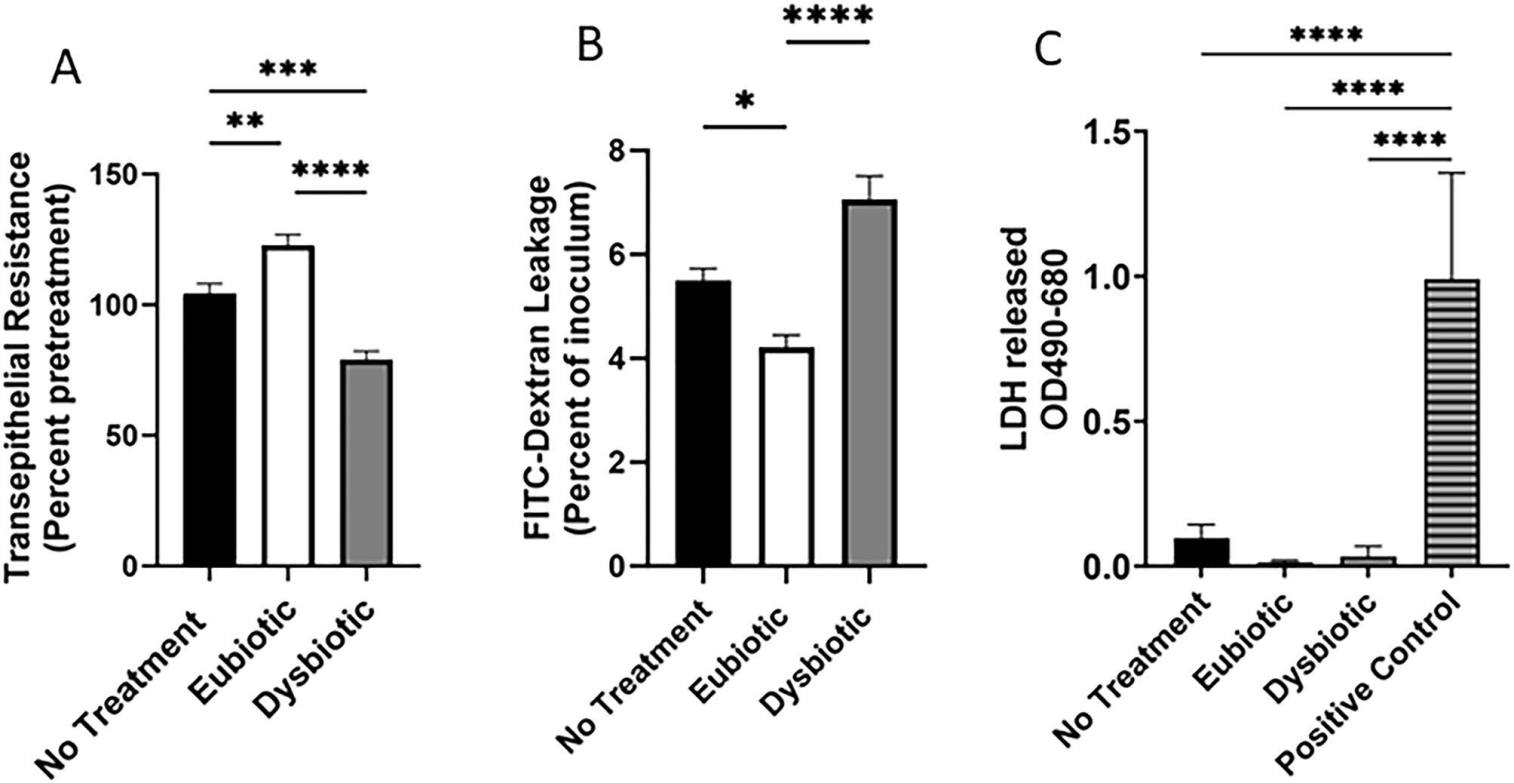
Treatment with SCFA + LA in eubiotic and dysbiotic concentrations respectively increases and decreases barrier integrity. VK2 cells were grown in ALI conditions for 5 days when baseline TER measurements were measured and media with and without eubiotic or dysbiotic concentrations of SCFA + LA was added to the apical side. (**A**) TER measurements were taken after 24 h of SCFA + LA treatment and reported as a percent of pre-treatment TER. (**B**) After 24 h of SCFA + LA treatment, media supplemented with 10 kDa FITC-dextran (2.4 mg/mL) was added to the apical side of the VK2 cell culture and after 24 h of incubation the basolateral media was collected and assessed for FITC-dextran leakage. (**C**) Apical media was collected after 24 h of SCFA + LA treatment and assessed for LDH concentration as a measure of cell cytotoxicity. Positive control indicates complete lysis of cells with lysis buffer. Data shown is a combined data from 3 individual experiments mean ± SEM with conditions done in triplicates. Statistical significance: *p < 0.05, **p < 0.01, ***p < 0.001, ****p < 0.0001.

Following a 24 h eubiotic treatment of VK2 cells, a significant increase in TER measurements indicating enhancement in barrier integrity, whereas dysbiotic treatment decreased TER measurement when compared untreated cells (Fig. 1A). Given the impact of both treatments on epithelial monolayer integrity, we next examined cell permeability by measuring leakage of FITC-dextran dye across the epithelial layers. In concurrence with the changes in barrier integrity, eubiotic conditions resulted in a significant decrease in permeability, while dysbiotic conditions exhibited a significant increase relative to untreated cells as shown by the leakage of the dye from apical side into the basolateral media (Fig. 1B). To ensure the changes in barrier integrity were not a result of effects of SCFA + LA treatment on cell viability, lactate dehydrogenase (LDH) was measured in the apical supernatants of VK2 cells following each treatment, and no statistically significant increase occurred, which indicated no decreased cell viability (Fig. 1C).

Next, a time course with dysbiotic and eubiotic treatment of epithelial cells was evaluated to determine if changes in barrier integrity were sustained over time, whereby TER measurements were taken every 24 h for up to 72 h. Notably, a time dependent enhancement of barrier integrity was seen under eubiotic conditions without causing cytotoxicity to the cells (Supplementary Fig. 1).

**Figure 2:**
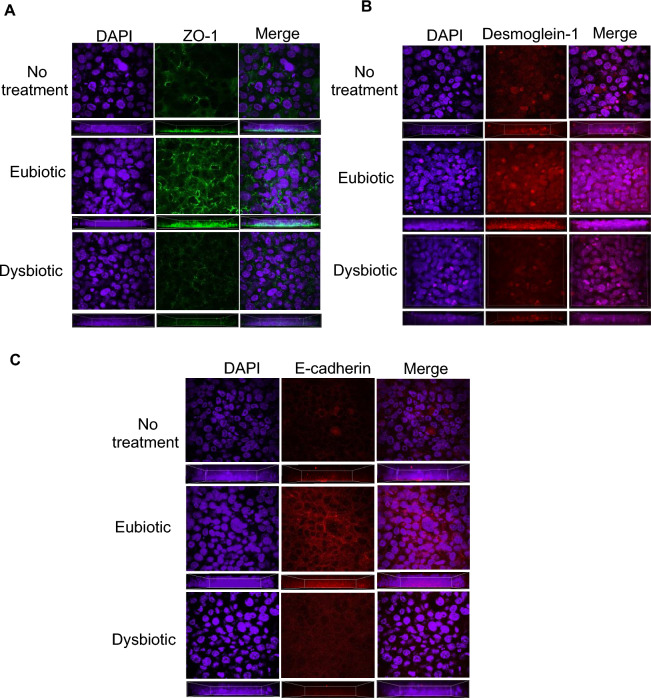
Eubiotic SCFAs treatment increased expression of cell structural adhesion proteins in vaginal epithelial cells. VK2 cells were grown in ALI conditions for 5 days when eubiotic or dysbiotic SCFA + LA containing media was added to the apical side. Cells were fixed after 24 h of SCFA + LA incubation and stained for (**A**) ZO-1 (green), (**B**) desmoglein-1 (red) and (**C**) E-cadherin (red) counterstain for nuclei with DAPI (purple) and visualized using confocal microscopy. Representative images are shown. N = 3. Magnification × 600. Images are presented as either *en face* composite *Z*-stack to illustrate the distribution of tight junction proteins or as a side view transverse profile of stacked Z- images below each panel to show the distribution of junctional proteins throughout the layers.

Considering the critical role of cell–cell structural adhesion proteins and their role in maintaining the epithelial barrier integrity^36^, their expression was measured following treatment conditions. Specifically, VK2 cells were incubated in eubiotic and dysbiotic conditions for 24 h, and then stained for the presence of ZO-1, a tight junction protein (Fig. 2A), desmoglein-1, a desmosomal protein (Fig. 2B) and e-cadherin, an adherens junction protein (Fig. 2C). Elevated expression of ZO-1, desmoglein-1 and e-cadherin was found in cells treated with a eubiotic SCFA + LA metabolite mixture as compared to VK2 cells treated with dysbiotic SCFA + LA or no treatment, indicating that enhanced expression of cell structural adhesion proteins was likely the mechanism for enhanced barrier integrity in eubiotic condition.

### Dysbiotic metabolite conditions initiate pro-inflammatory responses in vaginal epithelial cells

**Figure 3:**
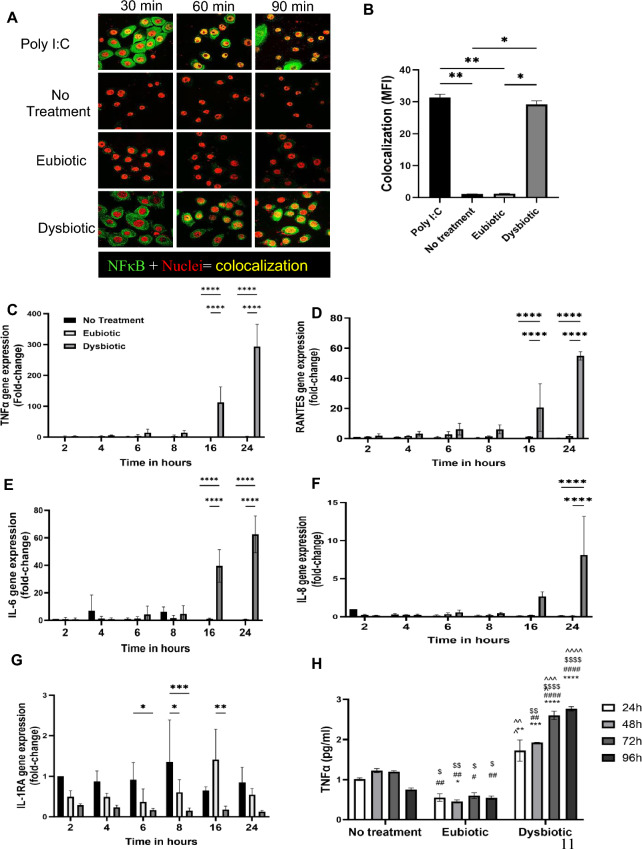
Dysbiotic SCFA conditions initiates an inflammatory response in vaginal Epithelial cells. VK2 cells were treated with either eubiotic or dysbiotic concentrations of SCFA + LA and fixed after 30, 60 and 90 min of treatments. Cells were stained for total NFκB p65 protein (green), while nuclei were stained red and visualized using Confocal microscopy. (**A**) The activation and nuclear translocation of NFκB is shown as colocalization of red nuclei and green NFκB resulting in yellow colocalization colour. Representative images are shown. Magnification × 600. (**B**) 6–9 images from each treatment at 90 min time point were analyzed for colocalized fluorescence by Image J software and presented as mean + /- SEM of fluorescence intensity of NFκB colocalization. (**C**–**H**) VK2 cells were grown as ALI cultures for 5 days before eubiotic or dysbiotic concentrations of SCFA + LA were added to the cell cultures and RNA was extracted at 2, 4, 6, 8, 16 and 24 h of SCFA + LA treatment and gene expression was determined by qRT-PCR for TNF-α (**C**), RANTES (**D**), IL-6 (**E**), IL-8 (**F**), IL-1RA (**G**). The fold change in each cytokine was expressed as the fold-change from no treatment results at 2 h of incubation. (**H**) TNF-α protein was also measured by ELISA in the supernatants collected after 24, 48, 72, and 96 h of treatment to confirm the qPCR results. Statistical comparisons are indicated with the following symbols: * compared with 24 h no treatment group; # compared with 48 h no treatment group; $ compared with 72 h no treatment group; ^ compared with 96 h no treatment group. Combined data from 3 individual experiments done in 3 technical replicates in each experiment. Statistical significance shown with different numbers of symbols: e.g. * *p* < 0.05, ** *p* < 0.01, *** *p* < 0.001, **** *p* < 0.0001.

Increases in the production of pro-inflammatory cytokines in female genital epithelial cells has previously been shown to disrupt barrier integrity, consequently increasing the risk of HIV transmission across the genital mucosa^18^. Given the decreased barrier function in dysbiotic condition, we examined inflammatory cytokines in VK2 cells treated with eubiotic and dysbiotic mixtures of SCFA + LA (Fig. 3A). Following the treatment, cells were fixed and stained to examine nuclear translocation of NFκB, indicating activation of this pro-inflammatory pathway. Poly I:C was used as a positive control, as it has been previously shown to induce NFκB activation in female genital epithelial cells^37^. Under conditions mimicking vaginal dysbiosis, nuclear translocation of NFκB was observed at 60–90 min. In comparison, noNFκB nuclear translocation was seen in untreated cells as well as those treated with a eubiotic SCFA + LA mixture (Fig. 3A).

Importantly, NFκB is a transcription factor that induces the downstream production of many pro-inflammatory responses ^18^. Therefore, we examined the effect of eubiotic and dysbiotic conditions on mediators of inflammation. Quantitative real-time polymerase chain reaction (qRT-PCR) was used to measure cytokine and chemokine production at 2, 4, 6, 8, 16 and 24 h in VK2 cells incubated with the eubiotic and dysbiotic SCFA + LA treatments. Overall, the dysbiotic SCFA + LA mixture showed a significant upregulation in the gene expression of TNF-α at 16 h and 24 h (Fig. 3C). Additionally, the dysbiotic treatment enhanced the expression of genes for RANTES (Fig. 3D), IL-6 (Fig. 3E) and IL-8 (Fig. 3F) at 16 h and 24 h relative to both the no treatment control and eubiotic conditions. In contrast, gene expression of IL-1RA, an anti-inflammatory cytokine, was downregulated when compared to untreated cells and eubiotic treatment conditions (Fig. 3G). Both eubiotic and dysbiotic conditions did not affect the expression of IL-1α or IL-1β (data not shown). Since TNF-α directly influences epithelial barrier function, we confirmed that TNF-α protein was significantly upregulated in supernatants collected after 24–96 h of dysbiotic treatment (Fig. 3H).

### Individual SCFAs, but not LA, upregulate NFκB expression

**Figure 4:**
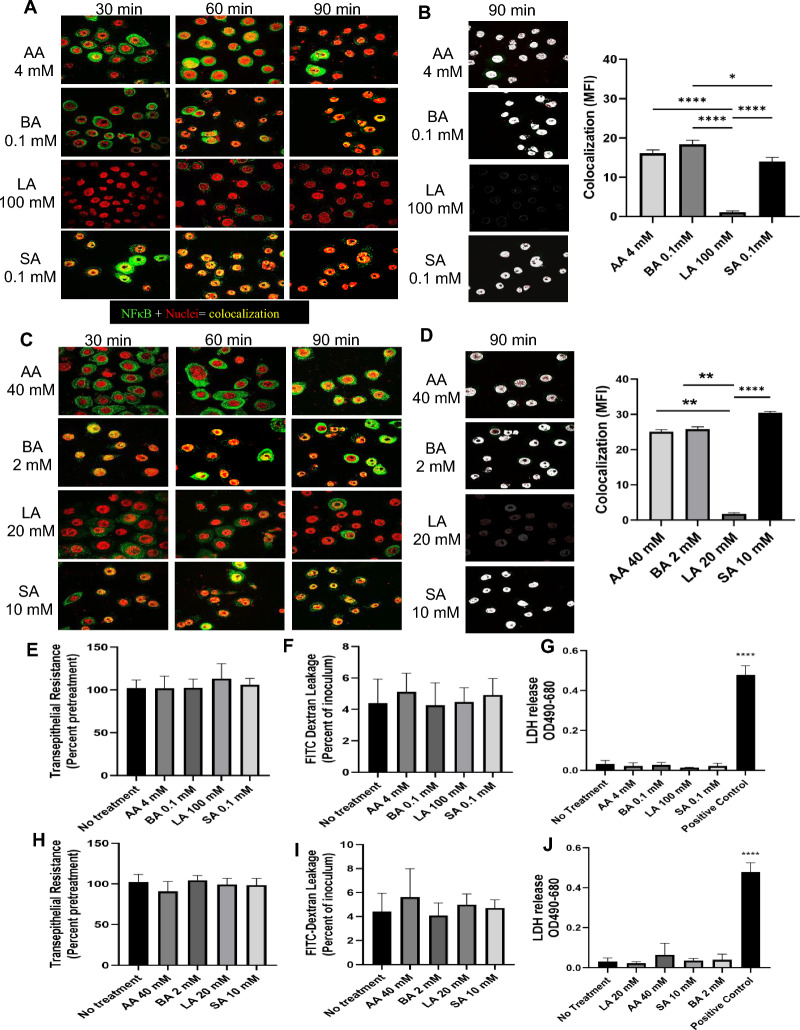
Eubiotic and dysbiotic concentrations of AA, SA and BA individually upregulate NFκB expression without causing changes in barrier integrity. VK2 cells were treated with eubiotic (**A**) and dysbiotic (**C**) concentrations of individual SCFA and LA. Cells were stained for NFκB. Representative immunofluorescence images of VK2 cells show NFκB (green) protein, nuclei (red) staining and translocation of NFκB into the nuclei (yellow) by confocal microscopy. Magnification × 600. (**B** and **D**) Colocalized pixel maps of NFκB stained images at 90 min post treatment are shown above in A and C were processed by overlapping the two colors green and red in each image by using ImageJ/FIJI software. Graphs shown (**B** and **D**) mean fluorescence intensity of colocalized pixels at 90 min time point. *p < 0.05, **p < 0.01, ****p < 0.0001VK2 cells that were grown for 5 days in ALI culture conditions before eubiotic (**E**–**G**) or dysbiotic (**H**–**J**) concentrations of SCFA and LA were added to the apical side of the cell layers. TER measurements were taken before treatment and after 24 h of eubiotic (**E**) or dysbiotic (**H**) SCFA and LA incubation and expressed as a percent of pre-treatment TER. After 24 h of treatment with eubiotic (**F**) and dysbiotic (**I**) SCFA and LA, media supplemented with 10 kDA FITC-dextran (2.4 mg/mL) was added to the apical side of the VK2 cell culture and after 24 h of incubation the basolateral media was collected and assessed for FITC-dextran leakage. Apical supernatants were also collected after 24 h of VK2 ALI cultures treated with eubiotic (**G**) or dysbiotic (**J**) individual concentrations of SCFA and LA and assessed for LDH concentration. Positive control with complete cell lysis, using a cell lysis buffer, showed significant high amounts (****p < 0.0001) of LDH release as compared to all other treatments that showed little or no release and no significant difference between each other. Data shown represents mean ± SEM (n = 3) with conditions done in 3 technical replicates in each experiment.

Having shown the pro-inflammatory response in the context of a mixture of metabolites at dysbiotic concentrations, we next examined the role of individual metabolites. VK2 cells were treated with individual SCFAs (AA, SA, BA) and LA at same concentrations they were present in vaginal eubiotic and dysbiotic mixtures, then stained for NFκB. Treatment with AA, SA and BA but not LA at both eubiotic and dysbiotic concentrations showed nuclear translocation of NFκB (Fig. 4A–D). The colocalization pixels images to show translocation of NFκB into the nucleus showed colocalization in AA, BA and SA but not in LA (Fig. 4B and D). To examine the impact of individual metabolites on epithelial barrier integrity, TER and FITC Dextran leakage was measured following each treatment condition described above (Fig. 4E, F, H and I). No changes in TER or permeability were observed at both eubiotic and dysbiotic concentrations of individual metabolites (Fig. 4E, F, H and I respectively). Together this suggests that NFκB activation associated with individual SCFAs does not alter overall barrier integrity. No significant increases in LDH concentrations in the apical supernatant of cells treated with eubiotic (Fig. 4G) or dysbiotic (Fig. 4J) SCFAs and LA was observed. Additionally, no significant changes in TER (Figure S1, C–F) were seen over a 72 h time period, except for LA at 72 h at a concentration of 100 mM. Similarly, no changes in FITC-dextran leakage or LDH in the apical supernatants was seen following individual SCFA treatments over a 20–400-fold concentration range (results not shown).

### The protective effects of LA at a eubiotic concentration persists in the presence of dysbiotic SCFAs

**Figure 5:**
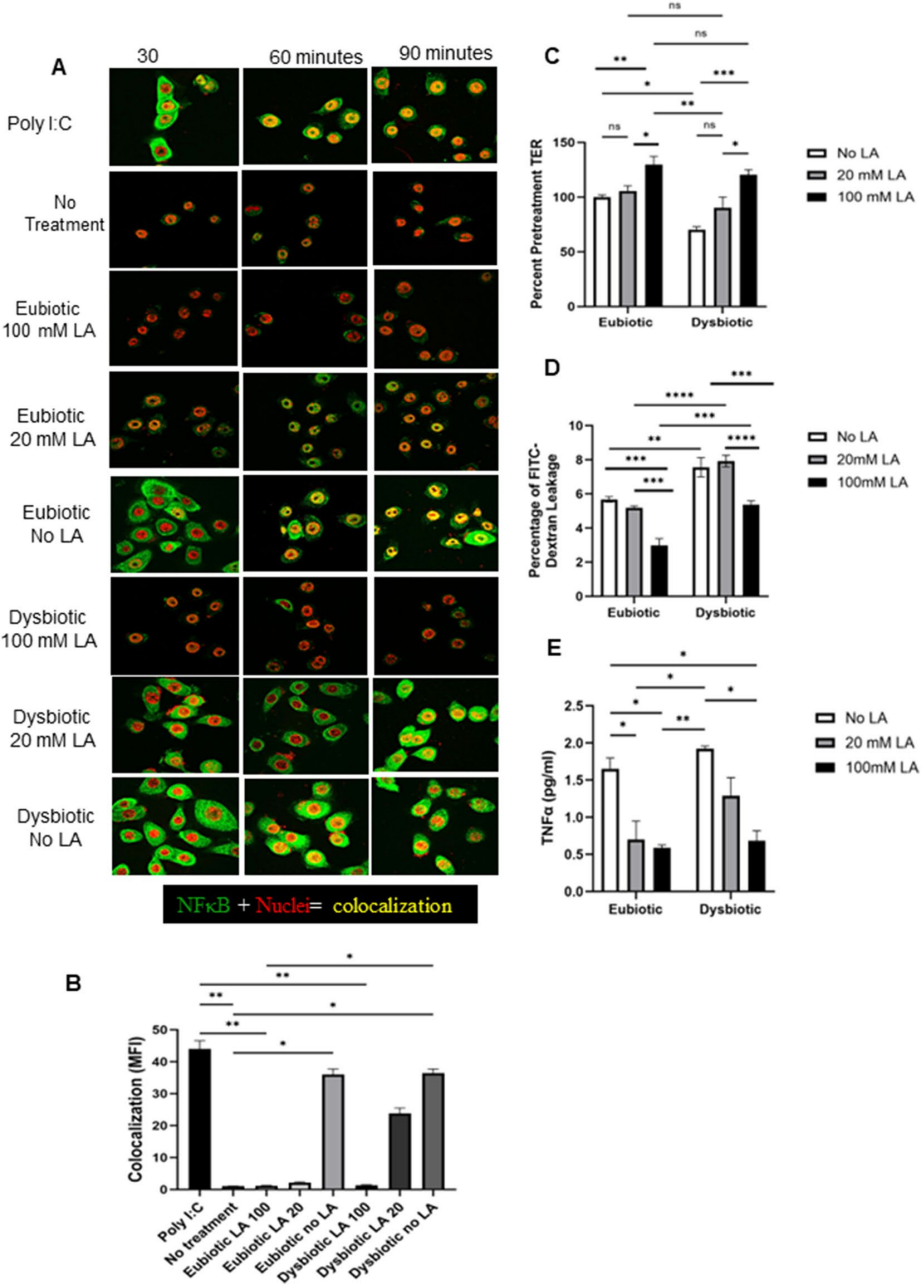
LA in higher concentrations ameliorates inflammation associated with dysbiotic SCFA conditions. (**A**) VK2 cells were treated with eubiotic and dysbiotic concentrations of SCFAs with no LA, eubiotic LA concentration (100 mM) or dysbiotic LA concentration (20 mM). Cells were fixed after 30, 60 or 90 min of treatment and stained for NFκB and images were captured under confocal microscope. Representative immunofluorescence images are shown. VK2 cells showed NFκB (green) protein, nuclei (red) staining and translocation of NFκB into the nuclei (red + green = yellow colocalization) by confocal microscopy. Magnification × 600. (**B**) Colocalized pixel maps of NFκB stained images at 90 min post treatment are shown in A were processed by overlapping the two colors green and red in each image by using ImageJ/FIJI software. Graphs shown mean fluorescence intensity of colocalized pixels at 90 min time point. (**C**) VK2 cells that were grown for 5 days in ALI culture conditions before eubiotic or dysbiotic concentrations of SCFAs with no LA, 100 mM LA concentration or 20 mM LA concentration was added to the apical side of the cells and TER was taken before and 24 h after incubation with treatments. (**D**) After 24 h of VK2 ALI cultures incubation with eubiotic or dysbiotic concentrations of SCFAs with no LA, 100 mM LA concentration or 20 mM LA concentration, media supplemented with 10 kDa FITC-dextran (2.3 mg/mL) was added to the apical side of the VK2 cell culture and after 24 h of incubation the basolateral media was collected and assessed for FITC-dextran leakage. (**E**) Supernatants were collected from VK2 cultures after 24 h of treatment with eubiotic or dysbiotic concentrations of SCFAs with no LA, 100 mM LA concentration or 20 mM LA concentration and subjected to TNFα ELISA to measure amount of TNFα produced. Data shown represents mean ± SEM (n = 3) with conditions done in 3 replicates in each experiment. Statistical significance: *p < 0.05, **p < 0.01, ***p < 0.001, ****p < 0.0001.

LA was the only metabolite tested that did not induce NFκB activation at either eubiotic or dysbiotic concentration levels, consistent with previous studies reporting its anti-inflammatory effects in human vaginal and cervical epithelial cells^38, 39^. To determines whether LA was suppressing NFκB activation in response to AA, SA and BA, VK2 cells were treated with a eubiotic metabolite mixture containing no LA, 20 mM LA (dysbiotic concentration) and 100 mM (eubiotic concentration) (Table 1). This was then repeated using a dysbiotic metabolite mixture containing no LA, 20 mM LA (dysbiotic concentration) and 100 mM (eubiotic concentration). Following NFκB staining, both activation and nuclear translocation was observed for eubiotic conditions with no LA present (Fig. 5A,B). NFκB activation was decreased when treated with a eubiotic metabolite mixture containing 20 mM LA similar to LA concentrations found during vaginal dysbiosis. This suggests that even at low concentrations LA can suppress NFκB activation, albeit to a lesser degree. Interestingly, although NFκB activation and nuclear translocation was exhibited in VK2 cells treated with dysbiotic conditions that did not include LA, no NFκB activation was revealed if the dysbiotic treatment was coupled with 100 mM of LA. Collectively, this data implies a direct role of LA in mediating anti-inflammatory responses, which is effective even in the presence of dysbiotic conditions.

We next examined given the anti-inflammatory effect of LA, if it could reverse the effect on barrier integrity and permeability. VK2 cells were grown in air liquid interface (ALI) cultures for five days and treated as described above, where eubiotic and dysbiotic mixtures were coupled with both no LA, 20 mM LA and 100 mM LA, following which TER was taken before and 24 h after treatment. Overall, a eubiotic concentration of LA (100 mM) was able to overcome the decrease in epithelial monolayer integrity mediated by dysbiotic metabolite concentrations, whereby TER values are within the same range of those measured following eubiotic treatment (Fig. 5B). Similarly, cell permeability was significantly diminished in the presence of 100 mM. LA relative to no LA and 20 mM LA in eubiotic and dysbiotic conditions (Fig. 5C).

Furthermore, TNF-α production was measured by ELISA, as a signature inflammatory marker in supernatants of VK2 cells following treatment, where LA at either 20 mM or 100 mM concentration diminished TNF-α production in VK2 cells with similar protein levels observed for 100 mM LA in the presence of eubiotic or dysbiotic concentrations (Fig. 5E). Overall, these data indicate that in addition to an anti-inflammatory effect, LA has protective effect on barrier integrity that endures even in the presence of dysbiotic SCFAs.

### Lactic acid is responsible for inducing and maintaining an acidic pH in the vaginal epithelial cultures

**Figure 6:**
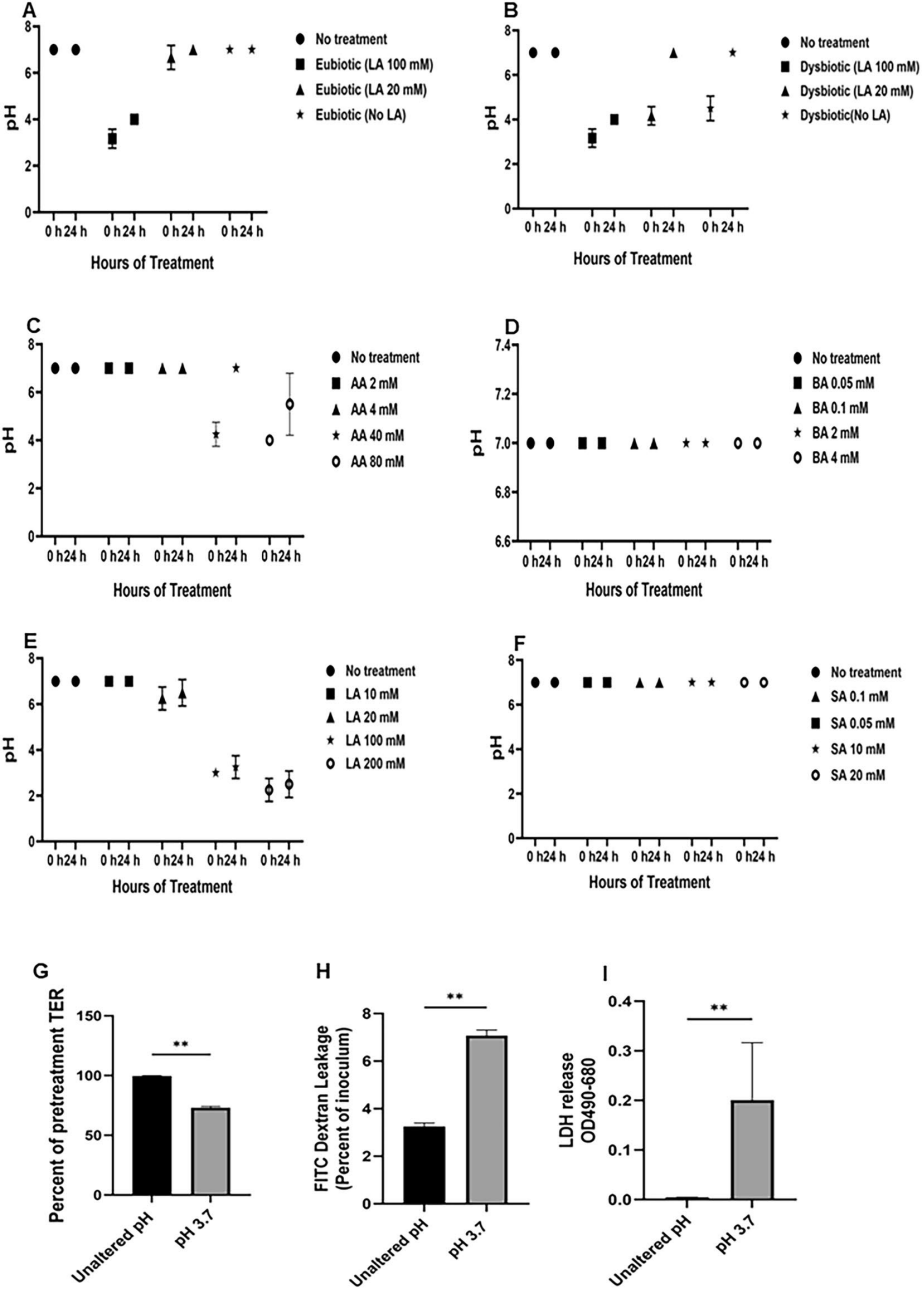
LA maintains an acidified environment in VK2 cell culture. VK2 cells were grown for 5 days in ALI culture conditions and eubiotic (**A**) or dysbiotic (**B**) concentrations of SCFAs with no LA, eubiotic LA concentrations or dysbiotic LA concentrations were added to the apical side of the cell layers and pH was measured just after adding the treatments and 24 h after incubation. VK2 cells that were grown for 5 days in ALI culture conditions and added with different concentrations of acetic acid (**C**), butyric acid (**D**), lactic acid (**E**) and succinic acid (**F**) to the apical side of the cell cultures and pH was measured before and 24 h after incubation. (**G**–**I**) VK2 cells were grown in ALI cultures for 5 days and ALI cultures were either unaltered or pH was altered using hydrochloric acid pH to 3.7. (**G**) TER was taken before and 24 h after treatment and presented as percent of pretreatment TER. (**H**) 24 h after altering the pH the treatment was removed and FITC dextran was added in the apical side and 24 h after basolateral supernatants were collected and analyzed for FITC dextran leakage. (**I**) The apical supernatants were also collected after 24 h of unaltered/altering pH to 3.7 to examine cytotoxicity by LDH assay. Data show represents mean ± SEM (n = 3) with conditions done in duplicates. Statistical significance: *p < 0.05, ****p < 0.0001.

The optimal vaginal microenvironment is characterized by microbiota and microbial products which help to maintain an acidic pH between 3.5 and 3.9. Previous studies have reported that at vaginal pH below its pKa of 3.9, LA can enhance barrier integrity^40, 41^. Therefore, we investigated the ability of individual SCFAs and LA to maintain an acidic pH below 3.9. pH of treated VK2 cells was measured in apical supernatants just after addition of treatments and after 24 h of incubation with individual SCFAs and LA (Fig. 6). As expected, a eubiotic metabolite mixture containing 100 mM of LA was able to acidify the media to a pH of 3 and maintain those conditions after 24 h (Fig. 6A). However, the same mixture with 20 mM LA was unable to lower the pH. Comparatively, when using dysbiotic SCFA concentrations in the presence of varying LA concentrations (no LA, 20 mM, and 100 mM), the pH of the media was initially acidified to a pH of 3.5–4 in all instances, however this could not be sustained over a 24 h incubation, except in the presence of 100 mM LA (Fig. 6B). To determine which metabolites in each mixture was contributing to the acidification, the pH of the apical supernatants was taken just after adding the treatments and after 24 h of incubation with individual concentrations of SCFA. Of the four metabolites only LA and AA were able to lower the pH to between 3 and 4 (Fig. 6C–F). However, AA could not maintain the acidic environment, even when repeated at concentrations upward of 80 mM (Fig. 6C). Importantly, while LA was able to both acidify and maintain an optimal acidic pH, a concentration of 100 mM was required (Fig. 6E) suggesting that a higher concentration is necessary for maintaining the protective properties of LA.

To determine if the effect of LA was specific or just lowering of pH could lead to similar protective effects, we used hydrochloric acid to lower pH to 3.7 which was equivalent of adding LA in 100 mM concentration in KFSM. The results showed that just changing the pH to 3.7 does not have any protective effect on the VK2 cells. In fact, the lowering of pH for 24 h was associated with significant deleterious effect on the epithelial ALI cultures, with significant lowering of TER (Fig. 6G), increased FITC dextran leakage (Fig. 6H) and significantly decreased the viability of the cells (Fig. 6I). This shows that the protective effects of LA are specific and not related to lowering of pH.

### Eubiotic metabolite concentrations ameliorate in HIV-1 mediated barrier leakage

**Figure 7:**
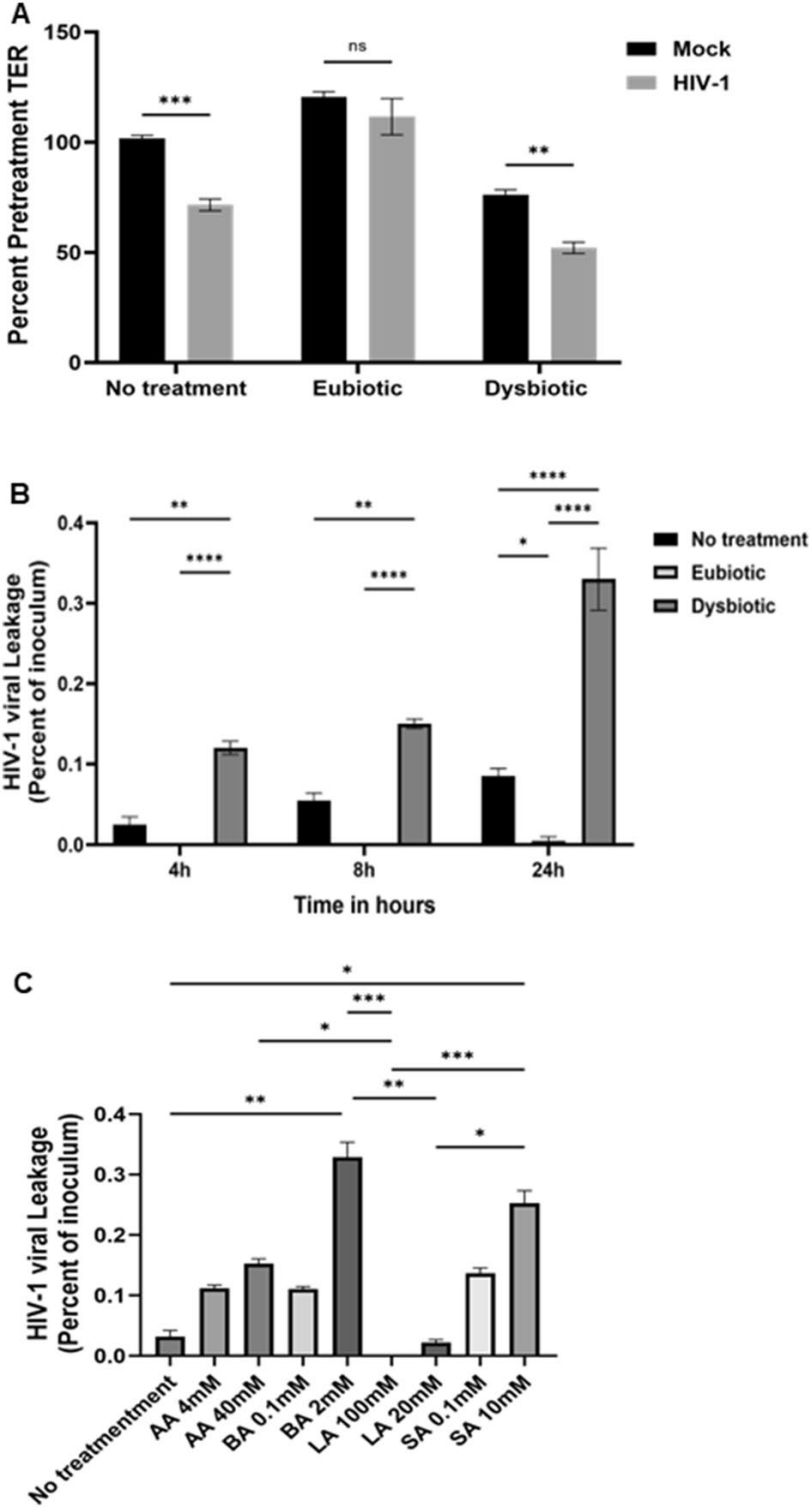
Eubiotic metabolite treatment reduced HIV-1 leakage through vaginal epithelial cells while dysbiotic metabolite treatment significantly increased HIV-1 leakage. VK2 cells were grown in ALI cultures, KSFM media with combination of eubiotic and dysbiotic concentrations of metabolites was added to the apical side. 24 h after treatments were added, the apical media with treatment was aspirated and HIV-1 (ADA, 10^5^ IU/well) was added on the apical side. (**A**) TER was measured before and 24 h post HIV-1 exposure in HIV-1 exposed and mock cultures and presented as percent pretreatment TER. (**B**) After 4, 8 and 24 h of HIV-1 exposure, the basolateral supernatants were collected and added to TZMbl cells. TZMbl cells were fixed and stained for β-gal activity in infected cells. The HIV-1 titers obtained from TZMbl cells were presented as percentage of HIV-1 inoculum. Data show represents mean ± SEM (n = 4) with conditions done in triplicates. (**C**) VK2 cells were exposed to eubiotic and dysbiotic concentrations of individual AA, BA, LA and SA for 24 h. After 24 h treatments were removed and HIV-1(ADA strain; 10^5^ IU/well) was added on the apical side. After 24 h of HIV-1 exposure basolateral supernatants were collected and HIV-1 was titred on TZMbl cells and presented as percent of inoculum. Statistical significance: *p < 0.05, **p < 0.01, ***p < 0.001, ****p < 0.0001.

Finally, we assessed if eubiotic or dysbiotic SCFA + LA mixtures contributed to a change in the HIV-1 leakage through the epithelial barrier. VK2 cells were grown in an ALI culture for 5 days, and 24 following treatments in eubiotic or dysbiotic conditions, HIV-1 ADA was added on the apical side and TER and viral leakage into basolateral side was measured. HIV-1 is known to decrease epithelial barrier function^18^, which was confirmed by significant decrease in TER in no treatment condition. Eubiotic condition did not induce the decrease TER in the absence of HIV-1 and even maintained the barrier function following exposure to HIV-1 (Fig. 7A). Dysbiotic condition decreased TER in the absence of HIV-1, which was further exacerbated following HIV-1 exposure.

Treatment with dysbiotic SCFA + LA mixture significantly increased HIV-1 leakage through the epithelial barrier compared to no treatment group at all time points, with maximum leakage observed after 24 h of HIV-1 exposure (Fig. 7B). In contrast, treatment with eubiotic SCFA + LA mixture significantly decreased HIV-1 leakage compared to no treatment condition and dysbiotic condition. These results show that eubiotic SCFA condition may help protect against HIV-1 infection by preventing its ability to cross the epithelial barrier while dysbiotic metabolite may increase HIV-1 susceptibility by increasing the amount of HIV-1 able to cross the leaky epithelial barrier in the FGT. To determine the effect of LA and individual SCFA, AA, BA and SA, they were added in eubiotic and dysbiotic concentrations to VK2 cells and the effect on HIV-1 leakage was measured (Fig. 7C). Only LA in both lower and higher concentrations did not increase or decreased the HIV-1 leakage across the VK2 cells, respectively, compared to media only controls. All other SFCAs, in low and high concentrations increased the leakage of HIV-1.

## Discussion

The physical and biochemical components modulating the mucosal barrier form the first line of defense against infection in the lower FGT^5, 42^. The interplay between microbiota, metabolites and immunity are critical in understanding susceptibility to STIs^29^. As such, this study was designed to investigate the role of SCFAs (SA, BA, AA) and lactic acid, which are immunomodulatory microbial metabolites, on inflammation and epithelial barrier integrity under conditions representative of vaginal eubiosis and dysbiosis. Our results demonstrated that metabolite treatment of VK2 cells under eubiotic conditions enhanced vaginal epithelial barrier integrity and increased the expression of cell–cell structural adhesion proteins, while dysbiotic metabolite treatment decreased barrier functions, activated NFκB, and up-regulated pro-inflammatory cytokines. When explored individually, SA, BA and AA at both eubiotic and dysbiotic concentrations resulted in an up-regulation of NFκB expression but LA consistently exerted an anti-inflammatory response. Furthermore, LA at higher concentrations (found in eubiotic conditions) was able to ameliorate the effects of dysbiotic condition on inflammation and barrier functions. The eubiotic condition also protected the barrier in presence of HIV-1 and decreased its leakage across vaginal epithelium. The results from this study add to the growing literature suggesting that LA should be assessed more closely as a prophylactic and/or therapeutic agent for bacterial vaginosis and possibly STIs^43^.

In terms of the chronology of events and timelines, based on our results, we would posit that the exposure to dysbiotic SFCAs leads to upregulation of NF-kB within 30–90 min (Figs. 3 and 4), followed by upregulation of inflammatory cytokine mRNA in 16-24 h and protein expression of TNF-a within 24 h (Fig. 3H). This inflammatory response leads to decreased expression of cell adhesion molecules, consequently resulting in decreased TERs, increased FITC dextran leakage and HIV-1 leakage measured at 24 h post-exposure (Figs. 1 and 7). The eubiotic mixture appears to induce anti-inflammatory gene IL-1RA expression in the same time frame (Fig. 3G) and addition of LA alone appears to suppress TNF-a production, leading to decreased inflammation and enhanced barrier functions (Fig. 5). A summary schematic summarizes this chronology of events under eubiotic and dysbiotic conditions (Supplementary Fig. 2).

Although previous literature has documented the pro-inflammatory response associated with dysbiotic concentrations of SA, BA and AA in genital epithelial cells^40, 42, 44, 45^, the impact of SCFA and LA on barrier integrity remains understudied. Recently, Delgado-Diaz *et. al.* examined effect of LA on gene expression of tight junction proteins and TER measurement in cervicovaginal epithelial cells and reported upregulation of both. Their previous study showed inflammatory effects of SCFA on cervicovaginal cells^40, 44^. Here we show for the first time the effects of both eubiotic and dysbiotic SCFA + LA mixtures on vaginal epithelium barrier functions and induction of inflammatory mediators which concurs with the clinical observations that eubiotic concentrations of SCFA + LA are protective, since they enhance epithelial barrier functions and induce anti-inflammatory factor IL-1RA^40^. In contrast the dysbiotic SCFA + LA mixture increased inflammatory mediators, including TNF-α and compromised barrier functions. When the effects of individual SCFA and LA on both inflammation and barrier function were examined, we concluded that LA was the primary factor that mediated the beneficial effects. Furthermore, LA could reverse the effects of dysbiotic mixture of SCFAs and HIV-1. To the best of our knowledge this study is one of the first to comprehensively examine this in a physiologically relevant model and demonstrate the functional benefits of LA to epithelial cells in clinical conditions.

The results from our study provide further support for testing LA as a therapeutic and/or prophylactic agent for promoting vaginal health. Lactic acid is a product of Lactobacillus species including *L. crispatus*, *L. gasseri* and *L. jensenii,* all of which are associated with optimal vaginal microbiota, and it plays an important role in lowering vaginal pH^32^. Both low pH and Lactobacillus that produce lactic acid are correlated with beneficial reproductive outcomes and lowered STI infectionsLA has been shown to inactivate BV-associated bacteria and pathogens such as *C. trachomatis*, HIV-1, and *N. gonorrhea*^41, 46–48^. It has also been shown to be anti-inflammatory and upregulate tight junction genes in cervicovaginal epithelial cultures^44^. Studies done in gut epithelium showed that the protective effect of lactic acid bacteria on barrier function was mediated by TLR2 and protein kinase C^49^. A recent systematic review concluded that while multiple clinical trials have been conducted on LA containing products there is a lack of high-quality evidence to support use of lactic acid for BV cure or microbiota modulation^43^. More rigorous randomized trials need to be done to evaluate its efficacy clinically. Our data suggests corroborates this by indicating that LA may be able to reverse the effects of SCFAs associated with dysbiotic conditions to decrease inflammation and protect barrier. It may also protect the vaginal barrier against HIV-1.

Our findings indicate that SA, BA and AA concentrations within a dysbiotic vaginal microenvironment induce a pro-inflammatory response through activation and nuclear translocation of NFκB. This was recapitulated both when VK2 cells were treated with these metabolites individually and as a mixture. Surprisingly, SA, BA and AA exerted an inflammatory effect regardless of the concentration. However, the induction of inflammatory mediators did not significantly decrease cell monolayer integrity when VK2 cells are treated with metabolites individually, implying that specific SCFAs must act synergistically to cause damage to the mucosal barrier. Together, these findings emphasize the critical role of AA, BA and SA during dysbiotic conditions in NFκB activation and pro-inflammatory responses, which in turn can result in a reduction in barrier integrity when metabolites are introduced as a mixture.

Our findings also align with clinical reports, as cervicovaginal lavages of BV cases, as well as incubation with BV-associated microbes like *G. vaginalis* induce NFκB activation and pro-inflammatory responses through likely through TLR-2 stimulation^13, 50^. Cell-free supernatants of G. vaginalis are also able to induce NFκB activation and pro-inflammatory responses, suggesting that bacterial secreted factors are sufficient in initiating an immune response^17^. A recent multi-omics study shows bacterial products associated with non-*Lactobacillus* dominant microbiomes activate the mammalian target of rapamycin (mTOR) pathway in vitro*,* which was associated with epithelial barrier disruption^51^. In clinical proteomic and transcriptomic datasets, non-*Lactobacillus* dominant microbiomes were significantly associated with both mTOR and NFκB activation^51^. In our studies, the gene expression of a number of pro-inflammatory factors like IL-6, IL-8, TNF-a and RANTES were upregulated in VK2 cells exposed to dysbiotic conditions, pointing to cumulative effects of multiple factors in barrier disruption. Overall, this suggests that dysbiosis in the FGT may contribute to epithelial barrier disruption through the activation of multiple inflammatory pathways. This has implications towards the mechanisms behind subclinical inflammation^52^, and HIV-1 susceptibility^18^.

Interestingly both 20 mM LA and 100 mM LA concentrations could modulate anti-inflammatory responses by diminishing TNF-α production and NFκB activation. The ability of LA to inhibit pro-inflammatory factors even in the presence of other SCFAs suggest this metabolite may be a significant contributor to the protective mechanisms associated with the *Lactobacillus spp*. Similarly, Delgado-Diaz et al.^40^ was able to show an 8.5-fold increase in the production of anti-inflammatory cytokine IL-1RA along with a reduction of IL-6, IL-8, IP-10, MIP-3α and RANTES in ectocervical epithelial cells using a eubiotic mixture containing L-LA at a pH of 3.9^40, 44^. Our data revealed that a 100 mM LA concentration was able to enhance barrier integrity even in the presence of higher concentrations of dysbiotic SCFAs. The enhanced barrier integrity and overall protective capacity of LA is thought to be partly a result of the acidic vaginal milieu created by LA itself, enabling LA to remain in its protonated state^53^. However, just lowering the pH does not recapitulate the beneficial effects of LA (Fig. 6G–I). Correspondingly, the data in this study highlights the ability of LA to not only acidify its environment, but also maintain the pH of < 3.9 over 24 h, even in the presence of varying concentrations of AA, BA, SA. These finding suggest that the protective effects of LA are likely concentration and pH dependent, whereby a strong inverse relationship between vaginal pH and LA concentration has been previously reported, implicating LA as the primary contributor to obtaining a vaginal pH of 3.5^41^. In comparison, while AA alone was able to acidify the pH to 4 initially, the effect was not maintained after 24 h like due to AA being a smaller more protonated molecule with a higher dissociation constant (pKa ~ 4.8 compared to 3.9). However, AA has been reported to have much weaker antimicrobial properties than L ^32^, which has implications for STI susceptibility in BV patients as AA is significantly elevated relative to LA during B ^3, 24^. For instance, LA has been previously reported to have chlamydia-cidal properties at a pH <  ^47, 54^. Overall, LA at physiologically relevant concentrations (i.e. 100 mM) can mitigate the inflammation and epithelial barrier damage induced by AA, SA and BA. Consequently, our findings suggest protonated LA at 100 mM, has potential for future prophylactics, capable of maintaining an acidic pH in high-risk populations.

Our observations regarding alterations in leakage of HIV-1 correlates with eubiotic and dysbiotic condition correlates with in vivo observations where dysbiotic microbiome and BV are known to increase susceptibility to HIV-1. Our lab has previously demonstrated that exposing female genital epithelial cells to HIV or the HIV envelope glycoprotein gp120 leads to the increase in permeability that was mediated by the disruption of tight junctional molecules like ZO-1, occludin and claudin-1, -2 and -4^18^. In the current study we observed the same phenomenon of increased permeability and leakage of virus from apical to basolateral site when VK2 cells were exposed to HIV-1 alone. Interestingly treatment with eubiotic SCFA mixture improved the vaginal barrier and blocked HIV leakage. Conversely, dysbiotic SCFAs exacerbated the HIV-1 effect and significantly increased HIV-1 leakage. Further studies are needed to determine if lactic acid alone can reverse the HIV-1 leakage and the underlying mechanism.

In conclusion we have demonstrated the ability of SCFA + LA at eubiotic concentrations to enhance epithelial barrier integrity and decrease cell permeability, while dysbiotic SCFA + LA diminish the epithelial barrier integrity, likely in part due to the observed pro-inflammatory responses. This work confirms previously reported mechanisms by which LA ameliorates inflammation and for the first time demonstrates that LA is primarily responsible for anti-inflammatory effects and protecting epithelial barrier in the presence of dysbiotic SCFA and can protect against HIV-1 leakage across vaginal epithelium. Collectively these findings provide potential prophylactic strategies among populations at higher risk for STI acquisition.

## Methods

### Cell line maintenance

The VK2 E6/E7 (Vk2) vaginal epithelial cell line was provided by Dr. Raina Fichorova (Brigham and Women’s Hospital, Boston, MA, USA). This cell line (ATCC CRL-2616) was derived from normal human vaginal mucosal tissue and immortalized at passage three by transduction with the retroviral vector LXSN-16 E6/E7 in the presence of polybrene. The VK2 cells were grown and maintained in keratinocyte serum-free medium (KSFM; Life Technologies, Carlsbad, CA, US) supplemented with 0.1 ng/mL of human recombinant epidermal growth factor (EGF), 0.05 mg/mL of bovine pituitary extract (BPE), 0.4 mM CaCl_2_, and 100 units/mL penicillin–streptomycin (all from Life Technologies, Carlsbad, CA, US) at a temperature of 37°C in presence of 5% CO_2_ as described previously by Lee et al., 2016^29^.

### Eubiotic vs dysbiotic metabolite treatments

KSFM media was used for no treatment controls. For the treatments KSFM media was supplemented with, DL Lactic acid (Cat# L1250) and SCFAs including AA, SA and BA (all from Sigma Aldrich) at concentrations between 0.1 and 100 mM, either individually or as a mixture to model eubiotic and dysbiotic environments. Metabolite concentrations were selected according to previously reported clinical data. Accordingly, LA has been previously detected at 89–133 mM in the cervicovaginal fluid (CVL) of women with *Lactobacillus* dominance^23, 40^, while a 3–fivefold reduction in LA is commonly observed in women with BV where a median LA concentration of 19.9 mM was found^33^. Similarly, CVLs from women with BV provided AA estimates of 40 mM^3^. SA has been previously reported at concentrations up to 0.61 mM, with a median of 0.02 in clinical controls, as compared to concentrations between 0.02 and 21.9 mM, in BV patients where an average of 10.1 mM was detected^33^. In addition, physiologically relevant concentrations of BA reported in controls relative women diagnosed with BV was observed to be 0.045–0.228 mM^27^ and 2–4 mM^35^. A summary of the selected treatment conditions and the relevant clinical data are summarized in Table 1.

### Air–liquid Interface (ALI) Cultures

To generate an ALI model closely mimicking the physiological conditions of the lower FGT^29^, a total of 60,000 VK2 cells were seeded on 0.4 µm pore-sized transwell polystyrene inserts (Grenier Bio-One, North Carolina, USA) in complete KFSM growth medium containing supplemented metabolites (Table 1). After 24 h, media from the apical side was removed, while media on the basolateral side was replenished every 48 h for 5 days of culture. On day five metabolite treatments were added to VK2 cells with trans-epithelial resistance (TER) measurements taken both pre-treatment and 24 h post-treatment. TER was measured across the cell layers using a volt ohm meter (EVOM; World Precision Instruments, Sarasota, FL, USA). TER in the transwell cultures is expressed as a percentage of TER 24 h after treatment relative to baseline TER measurements.

### Fluorescein Isothiocyanate (FITC)-dextran dye assay for cell layer permeability

FITC-dextran (10 kDa; 2.4 mg/mL, Sigma-Aldrich) was added to the apical surface of VK2 cells after 24 h of metabolite treatment, as described elsewhere^30^. Following a subsequent 24 h incubation with FITC dextran 50 µL of the apical and basolateral medium were sampled and placed in duplicate in a 96 well plate. FITC fluorescence was then measured using a microplate reader (Spectra Max i3, Molecular Devices, Sunnyvale, CA, USA) at an excitation and emission wavelength of 490 nm and 520 nm respectively. The FITC-dextran leakage in the basolateral compartment is expressed as a percentage of FITC-dextran added to the apical compartment.

### Lactate Dehydrogenase (LDH) Assay

Following 24 h metabolite treatments, apical supernatant was collected to determine lactate dehydrogenase (LDH) as a marker of cell stress^55^. Collected samples were analyzed using an LDH kit according to manufacturer instructions. (Pierce, Thermo Fisher Scientific, Waltham, MA, USA).

### Immunofluorescent staining and confocal microscopy

Immunofluorescent staining was performed as described previously by Nazli et al.^18^. Briefly VK2 cells were fixed following the metabolite treatments, permeabilized and stained for ZO-1, desmoglein-1, E-cadherin and NFκB using specific antibodies including, mouse anti-human ZO-1 (ThermoFisher Scientific, Mississauga, Ontario, Canada), mouse anti-human desmoglein-1 (abcam biotechnology company; Toronto, Ontario, Canada), mouse anti-human E-cadherin (Invitrogen; Burlington, Ontario, Canada) and rabbit anti-human NFκB p65 (Santa Cruz Biotechnology Inc., Dallas, Texas, US) respectively. All samples were imaged on an inverted confocal laser-scanning microscope (Nikon Eclipse Ti2) using standard operating conditions (63 × objective, optical laser thickness of 1 µm, image dimension of 512 × 512, lasers: green 488 nm and red 594 nm and purple 405 nm laser lines). For each experiment, confocal microscope settings for image acquisition and processing were identical between controls and treated cells with three separate, randomized images obtained for analysis.

### Relative gene expression by quantitative real-time polymerase chain reaction (qRT-PCR)

VK2 cells were treated with SCFA + LA, and RNA was extracted at multiple time points following treatment using the RNAeasy Plus Mini kit (Qiagen)^23, 24, 27^. Relative quantitative RT-PCR was performed using two-step SYBR green assays and the target genes were amplified with the following specific primers (forward and reverse primers, respectively): human tumor necrosis factor (TNF-α, accession number NM_000594): 5′-ATCAGAGGGCCTGTACCTCA-3’ and 5’-GGAAGACCCCTCCCAGA TAG -3′; C-X-C motif chemokine ligand 8 (CXCL-8) or Interleukin 8 (IL-8, accession number NM_000584): 5′-AGGGTTGCCAGATGCAATAC-3’ and 5-CCTTGGCCTCAATTTTGCTA-3′; Interleukin 6 (IL-6, accession number NM_000600): 5′-TACCCCCAGGAGAAGATTCC-3’ and 5’-TTTTCTGCCAGTGCCTCTTT-3′; interleukin 1 beta (IL-1β, accession number NM_000576) 5′-GGGCCTCAAGGAAAAGAATC-3’ and 5’- TTCTGCTTGAGAGGTGCTGA-3′; interleukin 1 alpha (IL-1α, accession number NM_000575) 5′-AATGACGCCCTCAATCAAAG-3’ and 5’-TGGGTATCTCAGGCATCTCC-3′; C–C motif chemokine ligand 5 (RANTES or CCL5, accession number NM_002985) 5′-AGTCGTCTTTGTCACCCGAAA-3’ and 5’-TCTCCATCCTAGCTCATCTCCAA-3′; interleukin 1 receptor type 1 (IL-1RA, accession number NM_000877) 5′-AATCCAGCAAGATGCAAGCC-3’ and 5’-ACGCCTTCGTCAGGCATATT-3′ and interleukin 10 (IL-10, accession number NM_000572) 5’-AGGAACCTCCAGTCTCAGCA-3’ and 5’-CAAAATTGGCTTGCAGGAAT-3′; glyceraldehyde-3-phosphate dehydrogenase (GAPDH, accession number NM_002046) 5′-ACAGTCAGCCGCATCTTCTTTTGC-3′; 5′-TTGAGGTCAATGAAGGGGTC-3′. The reaction was performed with RT2 SYBR® Green qPCR master mix according to the manufacturer’s manual (Qiagen) using the StepOne Plus™ Real-Time PCR System (Thermo Fisher, Waltham, MA, USA). Samples were run in triplicate and all data was normalized to GAPDH gene expression as an internal control. Fold change in gene expression was calculated according to metabolite treated samples relative to no treatment controls.

### TNF-α ELISA

TNF-α protein was measured by using “Human TNF-α ultra-sensitive ELISA kit “(Invitrogen) according to manufacturer's instructions. Minimum detection limit is 0.09 pg/ml.

### HIV-leakage assay

VK2 cells were grown in an ALI culture for 5 days. On Day 5, baseline TER measurements were recorded. KSFM media with eubiotic (100 mM Lactic acid, 4 mM Acetic acid, 0.1 mM Succinic acid, 0.1 mM Butyric acid) and dysbiotic (20 mM Lactic acid, 40 mM Acetic acid, 10 mM Succinic acid, 2 mM Butyric acid) concentrations of metabolites was added to the apical side. 24 h after treatments were added, the apical media was aspirated HIV-1 strain ADA-dsred (10^5^ IU/ml) in 100 µL quantity was added on the apical side of the transwells. At 4, 8 and 24 h after HIV-1 exposure, the basolateral supernatants were collected and added to TZMbl cells (indicator cell line) seeded in 24 well plate. TZM-bl cell line was obtained from the NIH AIDS Research and Reference Reagent Program (Cat. no. 8129). The TZM-bl cell line is derived from a HeLa cell clone and was transfected to express CD4, CCR5 and CXCR4^56^ and also contain integrated reporter genes for firefly Luciferase and *E. coli* β-galactosidase under the control of an HIV-1 long terminal repeat^57^, permitting sensitive and accurate measurements of infection. 2 h after the addition of supernatants on TZMbl cell the cells were overlayed with 1 ml of DMEM medium supplemented with 10% of FBS. The plates were then incubated at 37° C for 48 h. After 48 h of incubation cells were fixed and stained for β-galactosidase activity. Blue HIV infected cells were counted, and leakage of HIV through treated cells in basolateral compartments was determined as percentage of inoculum.

### Statistical analysis

Statistical analysis and graphical representations were done using GraphPad Prism Version 9 (GraphPad Software, San Diego, CA, USA), where data is presented as the mean ± SEM from multiple experiments. Specifically, statistical significance (*p* < 0.05) was determined between treatments with a non-parametric one-way analysis of variance (ANOVA) using the Kruskal–Wallis test. The data presented with time course and dose responses passed the Shapiro–Wilk test for normal distribution and was analyzed by two-way ANOVA for comparing two independent variables with their respective control. A Dunn’s multiple comparison post-hoc test was also performed for pair-wise comparisons, with *p*-values indicated in the figure legends.

### Supplementary Information

Supplementary Information.

## Data Availability

The datasets used and/or analysed during the current study available from the corresponding author on reasonable request.
