# Supplementary material for: Lactic acid enhances vaginal epithelial barrier integrity and ameliorates inflammatory effects of dysbiotic short chain fatty acids and HIV-1
Source: Sci Rep. 2023 Nov 16;13:20065. doi: 10.1038/s41598-023-47172-y (PMC10654711; doi:10.1038/s41598-023-47172-y)
Supplement: Supplementary file 1 — Supplementary Information. [file 41598_2023_47172_MOESM1_ESM.docx]

**Supplementary Figures:**

**A**

**C**

**B**

**D**

**F**

**E**

**Supplementary Figure 1: Eubiotic SCFA media increases TER while dysbiotic SCFA media decreases TER without causing cytotoxic effects in vaginal epithelial cells over an extended time range**. VK2 cells were grown in ALI conditions for 5 days when baseline TER measurements were taken and media containing eubiotic or dysbiotic SCFAs were added to the apical side. (A) TER measurements were taken every 24 h of SCFA treatment for 4 days and reported as a percent of pre-treatment TER. (B) Every 24 h of SCFA treatment, apical media was collected and assessed for LDH concentration as a measure of cell cytotoxicity. Data shown are mean *+* SEM (n=3). Statistical significance: *p < 0.05, ***p < 0.001, ****p < 0.0001.


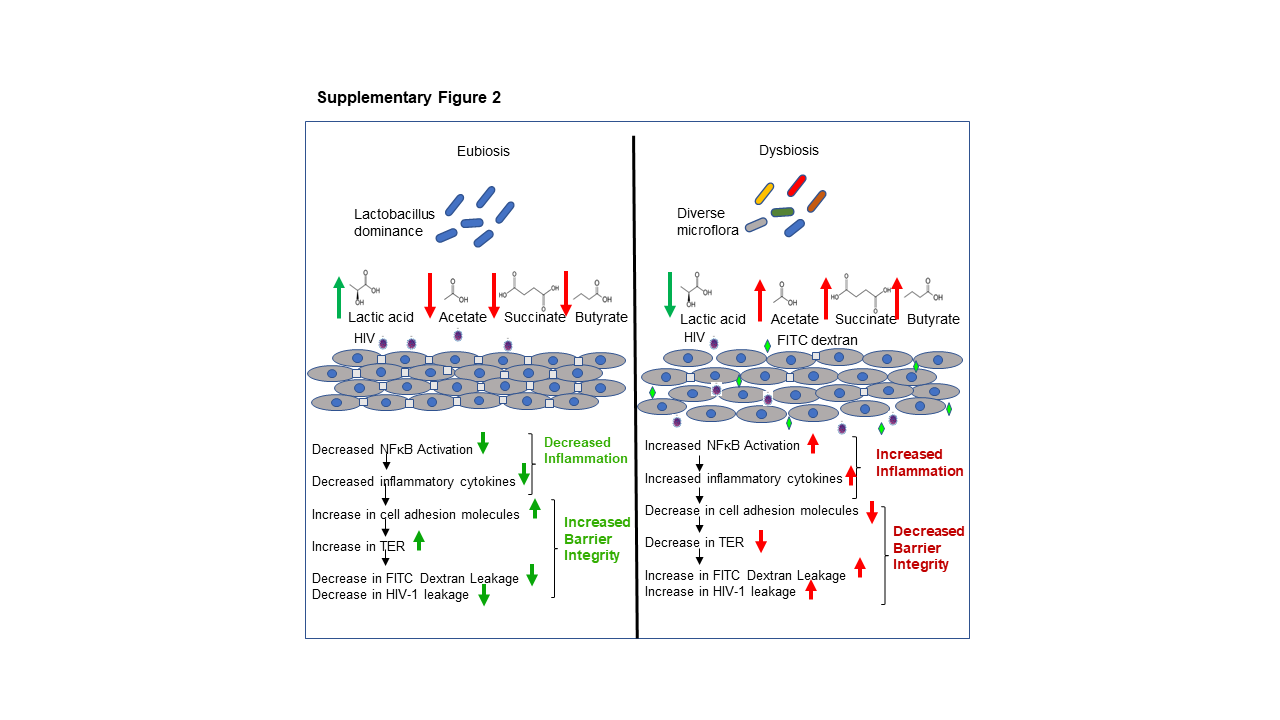


**Supplementary Figure 2:**

Lactic acid and short chain fatty acids (SCFA), acetic acid, succinic acid and butyric acid were added to vaginal epithelial cells in different concentrations to mimic eubiosis and dysbiosis in FGT. The barrier integrity was assessed by measuring transepithelial resistance (TER), FITC-dextran leakage, and expression of cell-to-cell adhesion molecules using immunofluorescent staining. Inflammation was assessed by examining gene activation and nuclear translocation of NFκB and gene expression of proinflammatory cytokines: TNFα, IL-6 and IL-8. HIV-1 leakage through vaginal epithelial cells was examined by measuring HIV-1 in basolateral compartment, post-treatment.
